# Effect of feeding bioactive compounds identified from plant extracts (4-hexylresorcinol, 7-hydroxycoumarin, and gamma-octalactone) on the productivity and quality of broiler meat

**DOI:** 10.14202/vetworld.2022.2986-2996

**Published:** 2022-12-30

**Authors:** Oleg Zavyalov, Duskaev Galimzhan, Kurilkina Marina

**Affiliations:** Federal Research Centre of Biological Systems and Agrotechnologies of the Russian Academy of Sciences, Orenburg, Russia

**Keywords:** chickens, liver, medicinal plants, pectoral muscles

## Abstract

**Background and Aim:**

Secondary bioactive compounds of medicinal plants exert anti-inflammatory, antimicrobial, antioxidant, and metabolism-modulating effects. This study aimed to investigate the effect of feeding 4-hexylresorcinol, as well as its combinations with gamma-octalactone and 7-hydroxycoumarin, on the digestibility of dietary nutrients, weight gain, and quality characteristics of the meat and liver of Arbor Acres broiler chickens.

**Materials and Methods:**

The following feeding scheme was applied on the chickens: Control, basal diet (BD); I experimental, BD + 4-hexylresorcinol at 0.5 mg/kg of live weight per day; II experimental, BD + 4-hexylresorcinol + gamma-octalactone at 0.4 mg/kg of live weight per day; III experimental, BD + 4-hexylresorcinol + 7-hydroxycoumarin at 0.1 and 0.15 mg/kg of live weight per day; and IV experimental, BD + 4-hexylresorcinol + gamma-octalactone + 7-hydroxycoumarin at 0.05, 0.15, and 0.01 mg/kg of live weight per day.

**Results:**

Chickens in I, II, and IV experimental groups at the age of 35 days showed superior live weight than chickens in the control group. Supplementation with all the tested additives, except the combination 4-hexylresorcinol + 7-hydroxycoumarin, significantly increased the digestibility coefficients of dietary nutrients. Supplementation with the combinations 4-hexylresorcinol + gamma-octalactone and 4-hexylresorcinol + gamma-octalactone + 7-hydroxycoumarin significantly increased the amount of fat in the pectoral muscles. However, the mass fraction of fat in the thigh muscles of broiler chickens decreased in II, III, and IV experimental groups. The pectoral muscles of broiler chickens in experimental Group IV contained small amounts of lysine, tyrosine, histidine, leucine–isoleucine, methionine, valine, proline, threonine, serine, alanine, and glycine. Supplementation with pure 4-hexylresorcinol significantly reduced the levels of lysine, phenylalanine, histidine, leucine–isoleucine, methionine, valine, proline, threonine, and alanine in the thigh muscles. However, supplementation with pure 4-hexylresorcinol significantly increased the concentrations of P, Fe, Se, Zn, and B and decreased the concentrations of I, Ni, V, Al, and Pb in the pectoral muscles. Supplementation with the combination 4-hexylresorcinol + gamma-octalactone + 7-hydroxycoumarin resulted in the accumulation of Ca, Co, Fe, Mn, Se, Zn, and Li and a decrease in the concentrations of K, Mg, and V.

**Conclusion:**

Supplementation with all the tested additives, except the combination 4-hexylresorcinol + 7-hydroxycoumarin, exerted a positive effect on the indicators of live weight gain and dietary nutrient digestibility in broiler chickens. Supplementation with the combinations 4-hexylresorcinol + gamma-octalactone and 4-hexylresorcinol + gamma-octalactone + 7-hydroxycoumarin increased the amount of fat in the pectoral muscles but decreased it in the thigh muscles. Supplementation with all the tested additives decreased the concentrations of I in the pectoral muscles and Zn in the thigh muscles in all the experimental groups compared with those in the control group.

## Introduction

Since the discovery and use of penicillin, antibiotics have played an unprecedented role in the prevention, control, and treatment of infectious diseases [1]. However, the indiscriminate use of antibiotics raises concerns about the development of resistant bacteria, which could result in the transfer of resistance factors from animals to humans [2]. Consequently, the need to develop possible alternatives to antibiotics has increased [3]. Recently, several researchers involved in studies of farm animal feeding have paid attention to the bioactive substances formed in plants [4]. It has been established that secondary bioactive compounds of plants improve the functions of the immune system, exert a significant impact on the health of animals, and increase productive qualities [5]. Among the factors that determine the relevance of the identification of secondary bioactive compounds of plants in animal husbandry are the fact that their global use as an alternative to various antimicrobials will not only protect animal health and increase animal productivity but also provide a solution to public health problems, including the problem of improving the safety of food products by eliminating the cumulative effects of antibiotics in animal products (milk and meat) [6, 7]. In animals with a monogastric stomach, these feed additives reduce the pathogenic load in the intestine, stimulating the formation of beneficial intestinal microbes and, as a result, increasing feed digestibility [8]. Some authors have reported that secondary bioactive compounds exert a positive effect on nutrient absorption by increasing the digestibility of crude protein [9–12]. In farm animals such as pigs, these compounds enhance nutrient absorption and the production of digestive secretions, reducing pathogenic stress in the gut [13]. Moreover, numerous studies have reported on the effect of active compounds contained in phytogenic feed additives on the productivity and quality of poultry products [14, 15]. In particular, the addition of the extracts of *Macleaya cordata* to the diet of laying hens increased the thickness of the eggshell [16], and adding the essential oils contained in the extracts of *Citrullus lanatus* to the diet of laying hens significantly increased the egg weight [17]. Furthermore, the addition of phytochemicals to feed increased the quality of carcasses and the yield of pectoral muscles in broiler chickens [18, 19]. Studies have also reported that these bioactive compounds increase the antioxidant status of meat [20–22] and exert a significant effect on the quantitative and qualitative composition of fatty acids in broiler breast muscles [23, 24].

Nevertheless, despite the relatively high level of knowledge about the use of secondary bioactive plant compounds in livestock and poultry, agricultural science is exploring new nutritional solutions that can ensure high levels of productivity and quality of meat products. Several herbal preparations have appeared on the veterinary market in recent years, including 4-hexylresorcinol. A wide range of the therapeutic properties of 4-hexylresorcinol in the human body and laboratory animals is associated with anti-inflammatory, antiseptic, analgesic, antibacterial, and immunomodulatory effects [25, 26]. Simultaneously, a limited number of studies have explored the potential use of this drug in the poultry industry [26]. Furthermore, there are almost no data on the effects of the combined use of 4-hexylresorcinol with other plant extracts, which could potentially enhance its positive effect on the physiological and productive indicators of poultry.

Therefore, this study aimed to investigate the effect of feeding 4-hexylresorcinol, as well as its combinations with gamma-octalactone and 7-hydroxycoumarin, on the digestibility of dietary nutrients, weight gain, and quality characteristics of the meat and liver of Arbor Acres chickens.

## Materials and Methods

### Ethical approval

The Local Ethics Committee of the Orenburg State University, Orenburg, Russia, approved the protocol of this investigation (№. 2022/11 dated February 10, 2022). All animal studies were performed following the ethical standards laid down in the 1964 Declaration of Helsinki and its later amendments.

### Study period and location

The study was conducted in March–April 2022 in the conditions of three poultry farms (Orenburgskaya Poultry Farm CJSC) located on the territory of the Orenburg Region, Russia.

### Experimental scheme

This study was conducted on 125 heads of 7-day Arbor Acres broiler chickens. The chickens were randomly divided into five groups with five replicates (cages) and five birds per replication. The feeding scheme was as follows: Control, basic diet (BD); I experimental, BD + 4-hexylresorcinol at 0.5 mg/kg of live weight per day; II experimental, BD + 4-hexylresorcinol + gamma-octalactone at 0.4 mg/kg of live weight per day; III experimental, BD + 4-hexylresorcinol + 7-hydroxycoumarin at 0.1 and 0.15 mg/kg of live weight per day; and IV experimental, BD + 4-hexylresorcinol + gamma-octalactone + 7-hydroxycoumarin at 0.05, 0.15, and 0.01 mg/kg of live weight per day.

Regarding the characteristics of the additives, 4-hexylresorcinol (propylresorcinol) and 7-hydroxycoumarin are small molecules identified in the extract of *Quercus cortex*, and gamma-lactone (gamma-octalactone) is a small molecule identified in the extract of *Eucalyptus viminalis*.

The following additives were purchased from Acros Organics (Belgium): 7-hydroxycoumarin (99% AC12111-0250, Acros), 4-hexylresorcinol – 2-n-propylresorcinol (98% AVH27024, Acros), and gamma-octalactone – gamma-octane lactone (97% ALO400-8, Acros). These additives were stored according to the manufacturer’s recommendations.

In the previous studies, a group of inhibitors of “quorum sensing” – plant metabolites used in this experiment – was identified [27], their toxicity was evaluated [28], and effective combinations were determined [29].

### Keeping and feeding technology

All birds were kept in three-tier battery cages in a room with temperature control and constant lighting. All broiler chickens had free access to feed and water. The room temperature was monitored daily and gradually reduced from 32°C to 24°C from day 0 to 21. The lighting program was set to produce 18 h of light and 6 h of darkness throughout the experimental period, and illumination was gradually reduced from 20 1× on day 0 to 5 1× on day 21. Moreover, all chickens received the Newcastle disease vaccine on day 7 and the inactivated infectious bursal disease vaccine on day 14. The content of nutrients and microelements in the primary diet of the examined poultry was within the limits of the needs of broiler chickens during the corresponding growing periods (Table-1) [30].

**Table-1:** The content of the basic diet [30].

Attributes	Starter (7–28 days)	Finisher (29–42 days)
	
	Control, I, II, and III	Control, I, II, and III
Ingredient composition (%)		
Wheat	48.0	44.0
Barley	3.2	0.9
Corn	10.0	21.0
Soybean meal (46% CP)	23.0	17.0
Sunflower meal (38% CP)	5.0	10.0
Sunflower oil	5.0	5.0
Dicalcium phosphate	1.6	1.6
Mel stern	0.9	1.5
Limestone	0.5	0.3
Salt	0.32	0.22
DL-Methionine	0.18	0.16
L-Lysine	0.35	0.17
Vitamin-mineral premixa^a^	2.0	2.0
Calculated nutrients Metabolizable energy (kkal/100 g)	298.0	305.0
CP	24.0	19.0
Methionine + cysteine	0.87	0.79
Lysine	1.35	0.96
Calcium	0.95	1.0
Available phosphorus	0.54	0.48

a Supplied following per kilogram of diet: Vitamin A: 7.000 IU, Vitamin D3: 800.0 IU, Vitamin E: 9 IU, Vitamin K3: 1.1 mg, Thiamin: 1.0 mg, Riboflavin: 5.0 mg, Vitamin B6: 2 mg, Vitamin B12: 0.05 mg, Vitamin C: 70 mg, Mn: 25 mg, Fe: 15 mg, Zn: 11 mg, Cu: 2.5 mg, I: 0.4 mg, Se: 0.5 mg, CP=Crude protein

### Evaluating the digestibility of dietary nutrients

The intake of feed into the body of experimental animals was calculated according to the data of daily accounting of consumption and weighing of residues. During the balance experiment (30–35 days of the experiment) conducted at the same time (morning and evening), poultry litter was collected. During the formation of the average sample, the litter was separated from the gravel and feather, and only after thorough mixing, the average samples were collected each day, which, when combined, constituted the average sample. Approximately 20%–50% of the homogenized mass of litter was collected each time in a jar with a ground-in lid. Manure nitrogen fixation was performed by adding a 0.1% solution of oxalic acid at the rate of 4 mL/100 g of manure. The amount of added oxalic acid was considered when calculating the initial moisture content in the litter. The collected portions of the litter were stored at 2°C–5°C. At the end of the reporting period, to determine the initial moisture content, the litter samples were dried at 60°C–70°C. The resulting air-dried mass was thoroughly ground and placed in a jar with a ground stopper. After conducting the balance experiments and performing analyses, the digestibility of individual nutrients was determined by calculating the actual average daily intake of feed nutrients and their excretion with litter per head.

### Slaughter of experimental birds and sampling

The experimental birds were slaughtered at the age of 35 days according to applicable recommendations [31]. Tissue samples of the chest, femoral muscle, and liver were collected immediately after slaughter and frozen at −18°C. The skin and subcutaneous fat were removed from the muscles used for trace element analysis.

### Amino acid analysis

The content of amino acids in meat was estimated by ion-exchange chromatography with postcolumn derivatization with a ninhydrin reagent and subsequent detection at a wavelength of 570 nm (440 nm for proline). The analyses were performed using a YL 9100 high-performance liquid chromatography (HPLC) System (Young Lin Instrument Co., Ltd, Korea) for HPLC, which consists of a YL9110 quaternary gradient pump, a YL9101 vacuum degasser, a YL9120 ultraviolet/visible detector, and a YL9150 autosampler (Pinnacle PCX post-column derivatizer, Na+ ion exchange column 4.0½150 mm, 5 mm, Na+ pre-column 3.0½20 mm, 5 mm; Pickering Laboratories, Inc., USA).

### Elemental composition analysis

The analysis of the elemental composition in the meat and liver of chickens included an evaluation of the concentrations of Ca, Cu, Fe, Li, Mg, Mn, Ni, As, Cr, K, Na, P, Zn, I, V, Co, Se, Ti, Al, Be, Cd, Pb, Hg, Sn, and Sr by inductively coupled plasma mass spectrometry (ICP-MS) and ICP atomic emission spectrometry on a Nexion 300D mass spectrometer (“PerkinElmer” USA) and Optima 2000 DV atomic emission spectrometer (“PerkinElmer”), respectively, in the laboratory of ANPO “Center for Biotic Medicine” (Moscow). Ashing was performed using a Multiwave 3000 microwave decomposition system (“AntonPaar,” Austria).

### Statistical analysis

The significance of differences was evaluated using the Mann–Whitney U test. The significance level (p) was considered at ≤0.05. Data were processed using the Statistica 10.0 software package (Stat Soft Inc., USA). Reference intervals were evaluated using the Reference Value Advisor for MS Excel (Microsoft Inc., USA).

## Results and Discussion

This study aimed to investigate the effect of feeding 4-hexylresorcinol and its various combinations with other plant extracts on the productive qualities of broiler chickens. Hence, we evaluated the dynamics of the live weight, as well as the absolute and average daily weight gains, of experimental birds in different growing periods (Table-2).

**Table-2:** The dynamics of live weight gains and safety of broiler chickens when biologically active compounds of medicinal plants are included in the diet.

Experimental period	Group				

	Control	I	II	III	IV
Beginning	182.0 ± 3.62	182.5 ± 7.04	182.0 ± 4.3	182.0 ± 4.42	182.6 ± 6.47
1 week	426.6 ± 9.34	437.5 ± 19.3	399.3 ± 18.9	431.6 ± 22.6	399.6 ± 19.2
2 week	852.3 ± 45.2	895.5 ± 38.1	820.6 ± 51.3	894.0 ± 45.6	838.6 ± 44.5
3 week	1497 ± 90.4	1596 ± 47.8	1443 ± 90.5	1561 ± 97.5	1487 ± 65.7
4 week	2079 ± 74.7	2255 ± 50.1	2100 ± 114.0	2190 ± 147.5	2148 ± 91.5
5 week	2605 ± 90.2	3023 ± 67.5^a^	2858 ± 76.3^a^	2893 ± 155.0	2931 ± 85.4^a^
Average daily weight gain	69.22 ± 10.2	81.1 ± 13.4	76.4 ± 13.8	77.4 ± 12.0	78.5 ± 14.3
Absolute gain	2423 ± 118.6	2841 ± 121.1^a^	2676 ± 92.1	2711 ± 101.2	2749 ± 125.3
Livability, %	92	100	96	96	100

I experimental – BD + 4-hexylresorcinol, II experimental - BD + 4-hexylresorcinol + gamma-octalactone, III experimental - BD + 4-hexylresorcinol + 7-hydroxycoumarin, IV experimental – BD + 4-hexylresorcinol + gamma-octalactone + 7-hydroxycoumarin.

a The difference is significant (p ≤ 0.05) in relation to the control group

Young chickens in I, II, and IV experimental groups at the age of 35 days showed superior live weight than chickens in the control group. An exception to the general positive trend in live weight gain was observed in young chickens that received the combination 4-hexylresorcinol + 7-hydroxycoumarin as part of the primary diet. Hence, despite the relatively high difference in live weight in the final growth period, the young chickens in experimental Group III showed no significant differences in this indicator compared with chickens in the control group, which was due to the high error of the arithmetic mean. Despite the significant differences in the live weight of experimental chickens at 5 weeks of age, up to 4 weeks of age, the growth rate in all experimental groups was approximately similar and was within the statistical error, which may indirectly indicate a cumulative and prolonged effect of the plant extracts on the body of broiler chickens. This assumption is based on the ability of some herbal preparations to accumulate in the body, initially without exerting a significant effect. However, their effect increases gradually. This effect is sometimes observed after the completion of supplementation exerting a long-term effect on metabolic processes.

Broiler chickens in experimental Group I, which received 4-hexylresorcinol as part of the primary diet, were characterized by the maximum intake of compound feed for the entire experimental period (Table-3).

**Table-3:** Feed intake and consumption by broiler chickens when biologically active compounds of medicinal plants are included in the diet, g/head.

Indicator	Group				

	Control	I	II	III	IV
Starter feed	1926	1971	1936	1807	1887
Starter feed	2129	2462	2406	2424	2464
Total per experiment	4055	4433	4342	4231	4351

I experimental - BD + 4-hexylresorcinol, II experimental - BD + 4-hexylresorcinol + gamma-octalactone, III experimental - BD + 4-hexylresorcinol + 7-hydroxycoumarin, IV experimental – BD + 4-hexylresorcinol + gamma-octalactone + 7-hydroxycoumarin

Regarding the digestibility of dietary nutrients, the chickens receiving pure 4-hexylresorcinol (experimental Group I) showed significantly better values than those in the control group in terms of the digestibility of dry matter, organic matter, crude protein, crude fiber, and nitrogen-free extractive substances, characterized by maximum values for these coefficients (Table-4). The broiler chickens fed with the combination 4-hexylresorcinol + gamma-octalactone + 7-hydroxycoumarin showed relatively lower values (although not significant) for the abovementioned coefficients than chickens in experimental Group I; however, the values were significantly greater than those of chickens in the control group. Similarly, adding the combination 4-hexylresorcinol + 7-hydroxycoumarin to the diet composition exerted no significant effect on the indicators of digestibility.

**Table-4:** Coefficients of digestibility of nutrients in the diet by broiler chickens when biologically active compounds of medicinal plants are included in the diet at the age of 30–35 days, %.

Experimental period	Group				

	Control	I	II	III	IV
Dry matter	73.76 ± 0.512	77.48 ± 0.671^a^	74.65 ± 0.07	73.90 ± 0.15	75.43 ± 0.11^a^
Organic matter	74.40 ± 0.502	78.03 ± 0.653^a^	75.24 ± 0.07	74.49 ± 0.15	75.98 ± 0.11^a^
Crude fat	69.99 ± 0.583	74.47 ± 0.521	71.54 ± 0.08^a^	70.16 ± 0.18	72.42 ± 0.13^a^
Crude protein	81.76 ± 0.352	85.17 ± 0.622^a^	82.52 ± 0.05	81.93 ± 0.11	83.12 ± 0.08^a^
Crude fiber	42.14 ± 1.12	50.13 ± 0.493^a^	45.83 ± 0.15^a^	43.66 ± 0.33	48.07 ± 0.24^a^
Nitrogen-free feed extractives	74.41 ± 0.504	77.74 ± 0.664^a^	75.02 ± 0.07	74.36 ± 0.15	75.69 ± 0.11^a^
Carbohydrates	72.28 ± 0.540	75.93 ± 0.644	73.10 ± 0.08	72.34 ± 0.16	73.88 ± 0.12^a^

I experimental – BD + 4-hexylresorcinol, II experimental – BD + 4-hexylresorcinol + gamma-octalactone, III experimental – BD + 4-hexylresorcinol + 7-hydroxycoumarin, IV experimental – BD + 4-hexylresorcinol + gamma-octalactone + 7-hydroxycoumarin.

a The difference is significant (p ≤ 0.05) in relation to the control group

The results of our experiment are generally consistent with those of the previous research [32]. This increase in the digestibility of dietary nutrients by the administration of bioactive compounds, including those identified from coumarin, may be due to the ability of such bioactive compounds to block quorum sensing (QS) signaling systems in the gastrointestinal tract and inhibit the formation of biofilms in pathogenic bacteria [33, 34]. This stimulates the growth of beneficial microflora, which results in complete absorption of nutrients by the chicken [35]. More recent research has also reported similar results, demonstrating the positive effect of the supplementation of gamma-octalactone isolated from *E. viminalis* leaf extract on the inhibition of various variants of broiler chicken QS systems [36].

To gain a complete understanding of the effect of the tested herbal preparations on the quality of meat products, we conducted a controlled slaughter of an experimental bird at the age of 35 days. The results of the morphological composition analysis of meat obtained from the broiler chickens are presented in Table-5.

**Table-5:** The chemical composition of muscles of broiler chickens when biologically active compounds of medicinal plants is included in the diet (35 days).

Indicator	Group				

	Control	I	II	III	IV
Pectoral muscles					
Moisture content, %	76.82 ± 3.01	78.03 ± 3.81	76.84 ± 4.24	78.66 ± 3.56	77.47 ± 3.44
Dry matter concentration, %	23.22 ± 0.982	22.01 ± 1.15	23.23 ± 1.25	21.48 ± 1.48	22.75 ± 0.776
Fat concentration, %	1.53 ± 0.0622	1.72 ± 0.0815	1.83 ± 0.0911^a^	1.35 ± 0.547	1.81 ± 0.0712^a^
Ash concentration, %	0.993 ± 0.0314	0.985 ± 0.0742	0.984 ± 0.044	0.995 ± 0.0512	0.983 ± 0.0624
Protein concentration, %	19.52 ± 0.621	18.48 ± 0.959	19.57 ± 0.795	17.94 ± 0.823	18.92 ± 0.902
Femoral muscles					
Moisture content, %	74.45 ± 2.02	78.32 ± 2.92	78.50 ± 3.54	82.93 ± 4.02	78.82 ± 3.63
Dry matter concentration, %	25.61 ± 1.49	21.77 ± 1.08^a^	21.50 ± 0.818^a^	19.12 ± 0.625^ab^	21.23 ± 0.791^a^
Fat concentration, %	3.10 ± 0.112	2.98 ± 0.179	2.61 ± 0.124^a^	2.52 ± 0.0612^ab^	2.72 ± 0.144^a^
Ash concentration, %	0.975 ± 0.0387	0.975 ± 0.0305	0.988 ± 0.0227	0.973 ± 0.0318	0.984 ± 0.0429
Protein concentration, %	21.65 ± 1.03	21.13 ± 0.969	20.37 ± 0.868	20.26 ± 0.945	20.44 ± 0.819

I experimental – BD + 4-hexylresorcinol, II experimental – BD + 4-hexylresorcinol + gamma-octalactone,

III experimental – BD + 4-hexylresorcinol + 7-hydroxycoumarin, IV experimental – BD + 4-hexylresorcinol + gamma-octalactone + 7-hydroxycoumarin. The difference is significant (p ≤ 0.05):

a the control group in relation to I, II, III, and IV, ^b^I group in relation to II, III, and IV, ^c^II group in relation to III and IV; ^d^III group in relation to IV

Adding the combinations 4-hexylresorcinol + gamma-octalactone and 4-hexylresorcinol + gamma-octalactone + 7-hydroxycoumarin to the diet of experimental birds significantly increased the fat content by 0.28% (p ≤ 0.05) and 0.30% (p ≤ 0.05), respectively, in the pectoral muscles compared with that in birds in the control group. However, the femoral muscles of broiler chickens in II, III, and IV experimental groups showed a significant decrease in fat content from 0.38% (p ≤ 0.05) to 0.58% (p ≤ 0.05). Although there was no significant effect of the tested additives on the dry matter content of the pectoral muscles, we detected a significant decrease in this indicator in all the experimental groups compared with the control group.

A comparative analysis of the amino acid composition of the meat of experimental groups showed that only supplementation with the combination 4-hexylresorcinol + gamma-octalactone + 7-hydroxycoumarin caused changes in the concentration of the primary amino acids in the pectoral muscles compared with that in the control group (Table-6).

**Table-6:** The content of amino acids in muscles of broiler chickens (35 days) when biologically active compounds of medicinal plants is included in the diet.

Indicator	Group				

	Control	I	II	III	IV
Pectoral muscles					
Arginine, %	5.61 ± 0.222	5.84 ± 0.194	5.91 ± 0.252	5.81 ± 0.265	5.13 ± 0.210^bcd^
Lysine, %	8.43 ± 0.354	8.25 ± 0.317	8.23 ± 0.291	8.54 ± 0.311	6.88 ± 0.437^abcd^
Tyrosine, %	4.58 ± 0.172	4.33 ± 0.118	5.02 ± 0.24^b^	4.89 ± 0.262^b^	3.76 ± 0.138^abcd^
Phenylalanine, %	3.41 ± 0.193	3.41 ± 0.0721	3.61 ± 0.188	3.62 ± 0.169	3.01 ± 0.123^bcd^
Histidine, %	2.92 ± 0.111	2.83 ± 0.0428	2.81 ± 0.139	3.01 ± 0.167	2.51 ± 0.129^abd^
Leucine-isoleucine, %	11.63 ± 0.372	11.61 ± 0.48	11.64 ± 0.467	11.92 ± 0.514	9.71 ± 0.452^abcd^
Methionine, %	2.53 ± 0.0824	2.52 ± 0.0559	2.54 ± 0.0727	2.52 ± 0.0918	2.23 ± 0.1021^abcd^
Valine, %	4.32 ± 0.284	4.13 ± 0.125	4.34 ± 0.262	4.30 ± 0.231	3.51 ± 0.178^abcd^
Proline, %	3.41 ± 0.154	3.43 ± 0.078	3.42 ± 0.169	3.44 ± 0.168	2.81 ± 0.127^abcd^
Threonine, %	4.33 ± 0.195	4.32 ± 0.118	4.31 ± 0.187	4.42 ± 0.173	3.51 ± 0.175^abcd^
Serine, %	2.74 ± 0.154	2.65 ± 0.0488	2.81 ± 0.162^b^	2.89 ± 0.137	2.24 ± 0.0932^abcd^
Alanine, %	7.64 ± 0.335	7.21 ± 0.268	7.84 ± 0.305	7.91 ± 0.277	6.31 ± 0.224^abcd^
Glycine, %	4.12 ± 0.163	4.20 ± 0.112	4.19 ± 0.169	4.21 ± 0.174	3.62 ± 0.172^abcd^
Femoral muscles					
Arginine, %	6.04 ± 0.251	5.52 ± 0.191	5.59 ± 0.238	6.30 ± 0.322^bc^	5.97 ± 0.269
Lysine, %	8.62 ± 0.374	7.12 ± 0.259^a^	7.93 ± 0.364	8.92 ± 0.413^b^	8.01 ± 0.342^b^
Tyrosine, %	3.73 ± 0.162	3.45 ± 0.172	3.54 ± 0.177	4.01 ± 0.218^b^	3.52 ± 0.115^d^
Phenylalanine, %	3.63 ± 0.152	3.02 ± 0.150^a^	3.35 ± 0.129	3.78 ± 0.162^b^	3.44 ± 0.154
Histidine, %	2.85 ± 0.133	2.21 ± 0.211^a^	2.50 ± 0.168	2.82 ± 0.149^b^	2.67 ± 0.126
Leucine-isoleucine, %	12.02 ± 0.525	9.94 ± 0.493^a^	11.32 ± 0.495^b^	12.61 ± 0.528^b^	11.47 ± 0.489^b^
Methionine, %	2.84 ± 0.092	2.12 ± 0.203^a^	2.59 ± 0.147^b^	2.92 ± 0.127^b^	2.65 ± 0.104^b^
Valine, %	4.32 ± 0.231	3.48 ± 0.135^a^	4.22 ± 0.173^b^	4.64 ± 0.192^b^	4.31 ± 0.210^b^
Proline, %	3.81 ± 0.164	3.02 ± 0.152^a^	3.68 ± 0.197^b^	3.87 ± 0.189^b^	3.64 ± 0.172^b^
Threonine, %	4.53 ± 0.147	3.64 ± 0.082^a^	4.27 ± 0.158^b^	4.53 ± 0.196^b^	4.12 ± 0.257
Serine, %	2.85 ± 0.174	2.43 ± 0.162	2.74 ± 0.148	3.01 ± 0.159^b^	2.73 ± 0.124
Alanine, %	7.02 ± 0.442	5.64 ± 0.185^a^	6.42 ± 0.381	6.9 ± 0.346^b^	6.64 ± 0.273^b^
Glycine, %	4.25 ± 0.234	3.73 ± 0.111	4.02 ± 0.194	4.13 ± 0.262	4.21 ± 0.224^b^

I experimental – BD + 4-hexylresorcinol, II experimental – BD + 4-hexylresorcinol + gamma-octalactone, III experimental – BD + 4-hexylresorcinol + 7-hydroxycoumarin, IV experimental – BD + 4-hexylresorcinol + gamma-octalactone + 7-hydroxycoumarin. The difference is significant (p ≤ 0.05):

a the control group in relation to I, II, III, and IV,

b I group in relation to II, III, and IV, ^c^II group in relation to III and IV,

d III group in relation to IV

In particular, the pectoral muscles of broiler chickens in experimental Group IV contained small amounts of lysine, tyrosine, histidine, leucine–isoleucine, methionine, valine, proline, threonine, serine, alanine, and glycine. Moreover, supplementation with pure 4-hexylresorcinol significantly reduced the levels of lysine, phenylalanine, histidine, leucine–isoleucine, methionine, valine, proline, threonine, and alanine.

The concentration range of the major nutrients determined in the biosubstrates was typical for these tissues and did not exceed the established standard values [37]. However, a comparative analysis of the data revealed the ambiguous nature of the effect of various combinations of 4-hexylresorcinol on the content of the major essential and toxic elements in the muscles of broiler chickens (Table-7).

**Table-7:** The content of chemical elements in the pectoral muscles of broiler chickens at the age of 35 days after the inclusion of biologically active compounds of medicinal plants in the diet.

Element	Group				

	Control	I	II	III	IV
Macroelements, g/kg					
Ca	0.0642 ± 0.0043	0.0961 ± 0.0347	0.0623 ± 0.0164	0.0502 ± 0.0054^a^	0.0474 ± 0.0083
K	3.98 ± 0.133	4.03 ± 0.123	5.28 ± 0.686	3.43 ± 0.0875^ac^	4.17 ± 0.233^d^
Mg	0.308 ± 0.0112	0.343 ± 0.0196	0.394 ± 0.0502	0.291 ± 0.0093^c^	0.319 ± 0.0072^d^
Na	0.652 ± 0.0114	0.629 ± 0.0231	0.605 ± 0.0913	0.972 ± 0.0385^ac^	0.540 ± 0.0314^d^
P	1.99 ± 0.0312	2.38 ± 0.0488^a^	2.59 ± 0.336	1.76 ± 0.0381^ac^	2.03 ± 0.108^d^
Essential trace elements, mg/kg					
Co	0.0032 ± 0.0024	0.0071 ± 0.0024	0.0063 ± 0.0044	0.0043 ± 0.0011	0.0054 ± 0.0035
Cr	0.329 ± 0.0351	0.311 ± 0.0572	0.416 ± 0.0685	0.357 ± 0.0055	0.375 ± 0.0553
Cu	0.397 ± 0.0235	0.554 ± 0.215	0.467 ± 0.0832	1.20 ± 0.731	0.368 ± 0.0443
Fe	10.09 ± 0.774	26.34 ± 6.86^a^	9.85 ± 1.31^b^	6.89 ± 1.21^ac^	9.96 ± 2.78
I	0.164 ± 0.0034	0.139 ± 0.0023^a^	0.141 ± 0.0104^b^	0.129 ± 0.0163^ac^	0.127 ± 0.0053^a^
Mn	0.176 ± 0.0042	0.166 ± 0.0155	0.198 ± 0.0335	0.139 ± 0.0062^a^	0.183 ± 0.0374
Se	0.164 ± 0.0101	0.257 ± 0.0325^a^	0.251 ± 0.0832	0.119 ± 0.0161^ac^	0.231 ± 0.0441
Zn	7.31 ± 0.173	9.46 ± 0.318^a^	7.73 ± 1.23	7.10 ± 0.117^c^	7.33 ± 0.577
Conditionally essential trace elements, mg/kg					
Li	0.0023 ± 0.0001	0.0024 ± 0.0004	0.0042 ± 0.0011	0.0023 ± 0.0014	0.0021 ± 0.0002
Ni	0.0172 ± 0.0034	0.0023 ± 0.0006^a^	0.0445 ± 0.0312	0.0342 ± 0.0121^c^	0.0182 ± 0.0057
Si	29.01 ± 5.27	37.82 ± 5.19	34.14 ± 5.49	26.50 ± 0.451^c^	29.99 ± 2.86
V	0.0322 ± 0.0040	0.0231 ± 0.0015^a^	0.0243 ± 0.0047	0.0362 ± 0.0021^c^	0.0233 ± 0.0034^d^
B	0.175 ± 0.0352	0.339 ± 0.0158^a^	0.214 ± 0.0292^b^	0.172 ± 0.0334^c^	0.190 ± 0.0413
Toxic elements, mg/kg					
As	0.0042 ± 0.0001	0.0037 ± 0.0005	0.0038 ± 0.0016	0.0047 ± 0.0001	0.0032 ± 0.0001
Al	1.42 ± 0.119	1.07 ± 0.128^a^	1.93 ± 0.299^b^	1.63 ± 0.393	1.72 ± 0.710
Cd	0.0001 ± 0.0001	0.0001 ± 0.0003	0.0001 ± 0.0001	0.0011 ± 0.0012	0.0012 ± 0.0013
High	0.0132 ± 0.0034	0.0053 ± 0.0032	0.0095 ± 0.0003	0.0083 ± 0.0045	0.0021 ± 0.0003^a^
Pb	0.0173 ± 0.0014	0,0125 ± 0.0017^a^	0.0154 ± 0.0045	0.0163 ± 0.0034	0.0197 ± 0.0072
Sn	0.0473 ± 0.0392	0.0075 ± 0.0013	0.0664 ± 0.0552	0.0103 ± 0.0035	0.0084 ± 0.0003
Sr	0.0483 ± 0.0062	0.0732 ± 0.0153	0.0523 ± 0.0092	0.0454 ± 0.0013	0.0485 ± 0.0157

I experimental – BD + 4-hexylresorcinol, II experimental – BD + 4-hexylresorcinol + gamma-octalactone, III experimental – BD + 4-hexylresorcinol + 7-hydroxycoumarin, IV experimental – BD + 4-hexylresorcinol + gamma-octalactone + 7-hydroxycoumarin. The difference is significant (p ≤ 0.05): athe control group in relation to I, II, III, and IV,

b I group in relation to II, III, and IV,

c II group in relation to III and IV,

d III group in relation to IV

The addition of pure 4-hexylresorcinol (experimental Group I) to the diet of experimental birds resulted in a significant increase in the concentrations of P, Fe, Se, Zn, and B and a decrease in the concentrations of I, Ni, V, Al, and Pb in the pectoral muscles, and its combined administration with 7-hydroxycoumarin resulted in a decrease in the concentrations of Ca, K, P, Fe, I, Mn, and Se and an increase in the concentration of Na. The inclusion of the complete combination of the tested bioactive compounds (4-hexylresorcinol + gamma-octalactone + 7-hydroxycoumarin) led to a significant decrease in the concentrations of I and Hg by 22.6% (p ≤ 0.05) and 84.1% (p ≤ 0.05), respectively. Supplementation with the combination 4-hexylresorcinol + gamma-octalactone exerted the minimum effect on the mineralization of the pectoral muscles of broiler chickens, manifested by a decrease in the concentration of only one essential element, iodine.

In a previous study, a positive trend in the accumulation of mineral elements in the edible parts of the bird’s body was demonstrated after the addition of plant components to the primary diet[38]. In our experiment, there was an ambiguous effect of the secondary dietary supplements of plant origin on the mineral composition of the femoral muscles (Table-8).

**Table-8:** The content of chemical elements in the femoral muscles of broiler chickens at the age of 35 days after the inclusion of biologically active compounds of medicinal plants in the diet.

Element	Group				

	Control	I	II	III	IV
Macroelements, g/kg					
Ca	0.0502 ± 0.0163	0.0604 ± 0.0072	0.0512 ± 0.0105	0.0894 ± 0.0123^bc^	0.113 ± 0.0222^abc^
K	4.01 ± 0.129	3.68 ± 0.138	3.79 ± 0.233	3.51 ± 0.156^a^	3.21 ± 0.237^a^
Mg	0.308 ± 0.0112	0.291 ± 0.0154	0.293 ± 0.0083	0.296 ± 0.0124^a^	0.275 ± 0.0110^a^
Na	0.669 ± 0.0171	0.694 ± 0.0234	0.643 ± 0.0393	0.576 ± 0.0262^b^	0.706 ± 0.0541^d^
P	1.97 ± 0.0373	1.76 ± 0.0445^a^	1.84 ± 0.0993	2.06 ± 0.0872^b^	1.83 ± 0.0893
Essential trace elements, mg/kg					
Co	0.0031 ± 0.0023	0.0042 ± 0.0012	0.0046 ± 0.0017	0.0052 ± 0.0027	0.0101 ± 0.0012^abcd^
Cr	0.353 ± 0.0165	0.299 ± 0.0359	0.457 ± 0.104	0.290 ± 0.0284	0.328 ± 0.0265
Cu	0.508 ± 0.126	0.733 ± 0.0577	0.798 ± 0.0778	0.490 ± 0.0251^bc^	0.692 ± 0.126
Fe	10.03 ± 0.806	18.65 ± 1.99^a^	34.80 ± 24.35	12.79 ± 2.47	16.93 ± 1.01^a^
I	0.151 ± 0.0151	0.0762 ± 0.0104^a^	0.109 ± 0.0071^ab^	0.214 ± 0.0262^abc^	0.169 ± 0.0123^bc^
Mn	0.180 ± 0.0062	0.181 ± 0.0084	0.329 ± 0.0991	0.137 ± 0.0173^abc^	0.210 ± 0.0072^abd^
Se	0.158 ± 0.0042	0.189 ± 0.0169	0.172 ± 0.0187	0.191 ± 0.0372	0.289 ± 0.0473^ac^
Zn	8.71 ± 1.57	17.14 ± 1.34^a^	16.40 ± 0.722^a^	12.79 ± 1.27^abc^	15.02 ± 0.779^a^
Conditionally essential trace elements, mg/kg					
B	0.130 ± 0.0372	0.245 ± 0.0363^a^	0.167 ± 0.0594	0.153 ± 0.0213^b^	0.161 ± 0.0334
Li	0.0021 ± 0.0002	0.0043 ± 0.0013	0.0022 ± 0.000	0.0031 ± 0.0017	0.0051 ± 0.0012^a^
Ni	0.0132 ± 0.0028	0.0113 ± 0.0051	0.0411 ± 0.0239	0.0012 ± 0.0001^b^	0.0012 ± 0.0001^b^
Si	30.89 ± 4.22	39.56 ± 2.25	33.01 ± 4.21	35.61 ± 4.31	35.46 ± 5.71
V	0.0322 ± 0.0041	0.0253 ± 0.0031	0.0212 ± 0.0021^a^	0.0190 ± 0.0022^a^	0.0219 ± 0.0013^a^
Toxic elements, mg/kg					
As	0.0041 ± 0.0002	0.0039 ± 0.0003^a^	0.0060 ± 0.0029	0.0021 ± 0.0001^b^	0.0039 ± 0.0008
Al	1.27 ± 0.171	1.697 ± 0.331	3.17 ± 1.17	1.83 ± 0.541	1.73 ± 0.232
Cd	0.0001 ± 0.0001	0.0001 ± 0.0000	0.0001 ± 0.0000	0.0001 ± 0.0000	0.0001 ± 0.0000
High	0.0101 ± 0.0058	0.0141 ± 0.0033	0.0022 ± 0.0001^b^	0.0123 ± 0.0024	0.0101 ± 0.0022
Pb	0.0231 ± 0.0015	0.0181 ± 0.0024a	0.0282 ± 0.0147	0.0242 ± 0.0109	0.0341 ± 0.0092
Sn	0.0474 ± 0.0392	0.0045 ± 0.0006	0.0203 ± 0.007^b^	0.0132 ± 0.0034	0.0164 ± 0.0086
Sr	0.0422 ± 0.0031	0.137 ± 0.0740	0.0581 ± 0.0072^a^	0.0333 ± 0.0061^bc^	0.0692 ± 0.0229

I experimental – BD + 4-hexylresorcinol, II experimental – BD + 4-hexylresorcinol + gamma-octalactone, III experimental – BD + 4-hexylresorcinol + 7-hydroxycoumarin, IV experimental – BD + 4-hexylresorcinol + gamma-octalactone + 7-hydroxycoumarin. The difference is significant (p ≤ 0.05):

a the control group in relation to I, II, III, and IV,

b I group in relation to II, III, and IV, ^c^II group in relation to III and IV,

d III group in relation to IV

Supplementation with 4-hexylresorcinol significantly increased the concentrations of Fe, I, Zn, and B and decreased the concentrations of P, As, and Pb in the femoral muscles. Supplementation with the combination 4-hexylresorcinol + gamma-octalactone resulted in the accumulation of Zn and Sr and slowed down the deposition of I and V. The effect exerted by 4-hexylresorcinol and 7-hydroxycoumarin involved reducing the deposition of K, Mg, Mn, and V in the femoral muscles, which was accompanied by an increase in the levels of I and Zn. Supplementation with the combination 4-hexylresorcinol + gamma-octalactone + 7-hydroxycoumarin exerted the maximum effect on the change in the mineral composition of the femoral muscles, manifested by the accumulation of Ca, Co, Fe, Mn, Se, Zn, and Li and a decrease in the concentrations of K, Mg, and V. Based on the previous studies, the reason for the increase in the concentrations of some chemical elements in muscle tissue samples in our study may be an increase in the conversion of feed minerals into meat due to the selective effect of the tested additives on beneficial intestinal microbiota during the functioning of the digestive system of birds [26, 39]. Moreover, the mechanisms of action of herbal supplements include altering the functions of the gastrointestinal tract and the induction of metabolic enzymes such as lipase and amylase. This increases the digestibility of feed minerals and stimulates appetite [40]. In addition, some studies have reported the effect of antagonism between the ions of some minerals and the presence of other chelating agents in chicken feed, which can act as competitors for the complex formation of minerals and affect the accumulation of these microelements in chicken meat [41, 42]. Considering certain aspects, the significant decrease in Ca concentration in the pectoral muscles of broiler chickens caused by supplementation with the combination 4-hexylresorcinol + 7-hydroxycoumarin can be explained by the formation of tannin–calcium complexes in the gastrointestinal tract of chickens, which is confirmed in the literature [43]. Moreover, it is difficult to explain the significant increase in the concentrations of several essential and toxic elements in the pectoral muscles with a simultaneous decrease in their content in the femoral muscles caused by consumption of the same diet. An obvious example of such a contradiction, in our experiment, is the significant increase in the concentration of I in the femoral muscles of broiler chickens in experimental Group III that received 4-hexylresorcinol along with 7-hydroxycoumarin along with a significant decrease in the concentration of I in the pectoral muscles. One study has described similar dynamics after feeding with the extract of *Boswellia serrata* to broiler chickens [44]. As an explanation for such an extraordinary regularity at first glance, that study provided data on the content of nutrients (dry matter, fat, and ash) in the pectoral and femoral muscles, which was confirmed by the evaluation of the morphological composition of those muscles in our experiment (Table-2). The authors of that study reported that the muscles of chicken breast and thigh differ significantly in their histochemical profile and the nature of metabolic processes, which can affect the mineral composition of meat. Therefore, a negative correlation was found between the muscles of the chest, lower leg, and liver and the Ca content and ether extract. This result can be explained by the fact that the compounds of Ca and fat in the gastrointestinal tract can form insoluble products, which, on the one hand, block the availability of Ca, and, on the other hand, impair fat absorption [45]. Furthermore, significant negative correlations were found between the contents of ash and P, Cu, and Fe [38].

In addition to the quality of meat products, the content of minerals in tissues relevant to consumption, for example, in offal, is examined. The liver is the most popular offal and widely used in cooking. Assessment of the concentrations of chemical elements in the liver of broiler chickens revealed the ambiguous nature of the effect of feed components on its elemental composition (Table-9).

**Table-9:** The content of chemical elements in liver tissue of broiler chickens at the age of 35 days after the inclusion of biologically active compounds of medicinal plants in the diet.

Element	Group				

	Control	I	II	III	IV
Macroelements, g/kg					
Ca	0,106 ± 0,0151	0,0858 ± 0,0110	0,0917 ± 0,0226	0,0769 ± 0,0194	0,0612 ± 0,0113^a^
K	3,28 ± 0,153	3,07 ± 0,168	3,26 ± 0,389	2,81 ± 0,227	3,03 ± 0,183
Mg	0,241 ± 0,0172	0,281 ± 0,0136	0,271 ± 0,0297	0,237 ± 0,0205	0,268 ± 0,0188
Na	1,00 ± 0,0433	1,22 ± 0,0563^a^	1,07 ± 0,133	1,12 ± 0,102	1,12 ± 0,0598
P	3,34 ± 0,187	3,11 ± 0,157	3,60 ± 0,394	3,13 ± 0,226	3,50 ± 0,215
Essential trace elements, mg/kg					
Co	0,0141 ± 0,0012	0,0267 ± 0,0016^a^	0,0215 ± 0,0036^a^	0,0173 ± 0,0029^b^	0,0192 ± 0,0048
Cr	0,384 ± 0,0794	0,393 ± 0,0297	0,481 ± 0,0552	0,350 ± 0,0303^c^	0,427 ± 0,0584
Cu	3,79 ± 0,284	4,89 ± 0,135^a^	5,53 ± 0,739^a^	4,43 ± 0,401	4,81 ± 0,226^a^
Fe	343,7 ± 15,45	273,3 ± 18,66^a^	269,7 ± 16,95^a^	225,3 ± 21,78^a^	214,0 ± 6,56^ac^
I	0,173 ± 0,0342	0,0732 ± 0,0055^a^	0,212 ± 0,0147^b^	0,126 ± 0,0104^bc^	0,130 ± 0,0197^c^
Mn	2,76 ± 0,162	2,87 ± 0,121	2,68 ± 0,313	2,09 ± 0,188^ab^	2,72 ± 0,144^d^
Se	0,671 ± 0,0762	0,860 ± 0,0328^a^	0,715 ± 0,0927	0,585 ± 0,0822^b^	0,670 ± 0,0486
Zn	31,35 ± 1,61	36,69 ± 1,74^a^	48,12 ± 5,77^a^	56,20 ± 5,97^ab^	34,89 ± 2,22^cd^
Conditionally essential trace elements, mg/kg					
B	0,1002 ± 0,0481	0,203 ± 0,0517	0,0703 ± 0,0304	0,0615 ± 0,0158^b^	0,138 ± 0,0252^d^
Li	0,0033 ± 0,0001	0,0039 ± 0,0007	0,0041 ± 0,0018	0,0038 ± 0,0017	0,0041 ± 0,0001
Ni	0,0032 ± 0,0022	0,0030 ± 0,0013	0,0091 ± 0,0068	0,0082 ± 0,0077	0,0031 ± 0,0023
Si	31,01 ± 2,05	38,56 ± 6,89	41,04 ± 4,64	29,32 ± 2,45^c^	42,36 ± 3,41^ad^
V	0,0402 ± 0,004	0,0531 ± 0,0062	0,0473 ± 0,0054	0,0391 ± 0,0047	0,0402 ± 0,0053
Toxic elements, mg/kg					
As	0,0051 ± 0,0012	0,0051 ± 0,0004	0,0052 ± 0,0011^b^	0,0043 ± 0,0018	0,0044 ± 0,0001
Al	1,38 ± 0,160	0,760 ± 0,0225^a^	1,58 ± 0,268^b^	0,956 ± 0,217	1,08 ± 0,216
Cd	0,0101 ± 0,0012	0,0263 ± 0,0012^a^	0,0150 ± 0,0011^b^	0,0121 ± 0,0010^bc^	0,0197 ± 0,0023^ad^
Hg	0,0092 ± 0,0023	0,0054 ± 0,0017	0,0102 ± 0,0039	0,0182 ± 0,0104	0,0082 ± 0,0022
Pb	0,0201 ± 0,0013	0,0121 ± 0,0007^a^	0,0223 ± 0,0034^b^	0,0213 ± 0,0064	0,0165 ± 0,0013^a^
Sn	0,0084 ± 0,0013	0,0041 ± 0,0012	0,0132 ± 0,0077	0,0032 ± 0,0014^a^	0,0053 ± 0,0038
Sr	0,0633 ± 0,0051	0,0964 ± 0,0059^a^	0,0671 ± 0,0054^b^	0,0683 ± 0,0076^b^	0,0632 ± 0,0064

I experimental - BD + 4-hexylresorcinol; II experimental - BD + 4-hexylresorcinol + gamma-octalactone; III experimental - BD + 4-hexylresorcinol + 7-hydroxycoumarin; IV experimental - BD + 4-hexylresorcinol + gamma-octalactone + 7-hydroxycoumarin. The difference is significant (p ≤ 0.05):

a the control group in relation to I, II, III, IV;

b I group in relation to II, III, IV;

c II group in relation to III, IV;

d III group in relation to IV

In general, it can be stated that 4-hexylresorcinol exerted the most pronounced effect on the concentration of chemical elements in the liver of experimental birds. The addition of 4-hexylresorcinol to the diet of broiler chickens in experimental Group I resulted in a significant increase in the concentrations of Na, Co, Cu, Se, Zn, Cd, Pb, and Sr and a significant decrease in the concentrations of Fe, I, and Al compared with those in the control group. In contrast, the addition of the combination 4-hexylresorcinol + gamma-octalactone increased the concentrations of Co, Cu, Fe, and Zn and simultaneously exerted no pronounced effect on the decrease in the concentrations of other elements. Supplementation with the combination 4-hexylresorcinol + 7-hydroxycoumarin significantly increased the levels of Zn, along with a significant decrease in the levels of Fe, Mn, and Sn. Supplementation with the combination 4-hexylresorcinol + gamma-octalactone + 7-hydroxycoumarin reduced the concentrations of Ca, Fe, and Cd and increased those of Cu and Si. In particular, the liver of broiler chickens consuming 4-hexylresorcinol-supplemented diet (experimental Group I) showed significantly elevated concentrations of several essential elements, but there was also a significant increase in the concentration of toxic elements (Cd and Pb). Considering that the liver plays an indispensable role in implementing the mechanisms of detoxification of the body because it acts as a bile producer [46], the increased content of toxic elements may be the result of their increased excretion from the body. An indirect confirmation of this assumption is the significantly low content of Pb in the pectoral and femoral muscles of broiler chickens in experimental Group I in our experiment.

## Conclusion

Bioactive substances found in the composition of plant extracts exert a positive effect on the live weight gain of broiler chickens. The absence of a significant difference at the initial stages of the experiment indicates a cumulative effect of feeding with the study substances on the body of broiler chickens. The addition of all the tested additives, except the combination 4-hexylresorcinol + 7-hydroxycoumarin, to the diet resulted in a significant increase in the digestibility coefficients of dietary nutrients. Supplementation with the combinations 4-hexylresorcinol + gamma-octalactone and 4-hexylresorcinol + gamma-octalactone + 7-hydroxycoumarin significantly increased the amount of fat in the pectoral muscles but decreased it in the femoral muscles. The addition of all the tested herbal preparations to the poultry diet resulted in a decrease in the concentrations of I in the pectoral muscles and that of Zn in the femoral muscles in all experimental groups compared with those in the control group. The observed trend of a decrease in the concentration of Pb in the pectoral and femoral muscles, along with an increase in the content of Pb in the liver, after supplementation with 4-hexylresorcinol, indicates an increase in the intensity of the detoxification process in the body.

## Authors’ Contributions

OZ: Mathematical processing of experimental data and drafted the manuscript. DG: Provided general guidance and developed experimental methods. KM: Conducted the experiments, analyzed, and interpreted the data. All authors have read and approved the final manuscript.
